# Supplementary material for: Red Light Irradiation In Vivo Upregulates DJ-1 in the Retinal Ganglion Cell Layer and Protects against Axotomy-Related Dendritic Pruning
Source: Int J Mol Sci. 2021 Aug 4;22(16):8380. doi: 10.3390/ijms22168380 (PMC8395066; doi:10.3390/ijms22168380)
Supplement: Supplementary file 1 [file ijms-22-08380-s001.zip › ijms-1242006-supplementary.pdf]

Supplementary Material

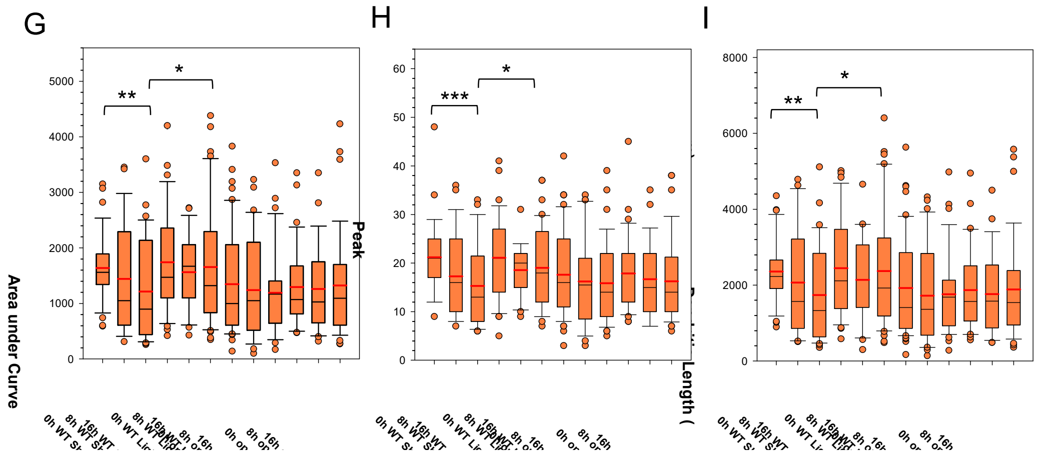

Supplementary Figure S1: Box and whisker plots corresponding to the bar charts shown in Figure 2 G, H and I.

(G) the area under the Sholl curve (AUC); (H) average maximum peak of Sholl curve; and (I) average total dendritic length of the entire population of RGCs. All measures revealed significant degeneration over 16 hours in the sham-treated WT RGCs which was inhibited by the light treatment, as shown by the 16-hour WT light group having significantly greater dendritic complexity. Black line: mean; Red line: median. The ends of boxes correspond to the 25th and 75th percentiles, the whiskers correspond to the 10th and 90th. * *p*<0.05, ** *p*<0.01, ***p<0.001, † *p*<0.05; Mann-Whitney *U* test.

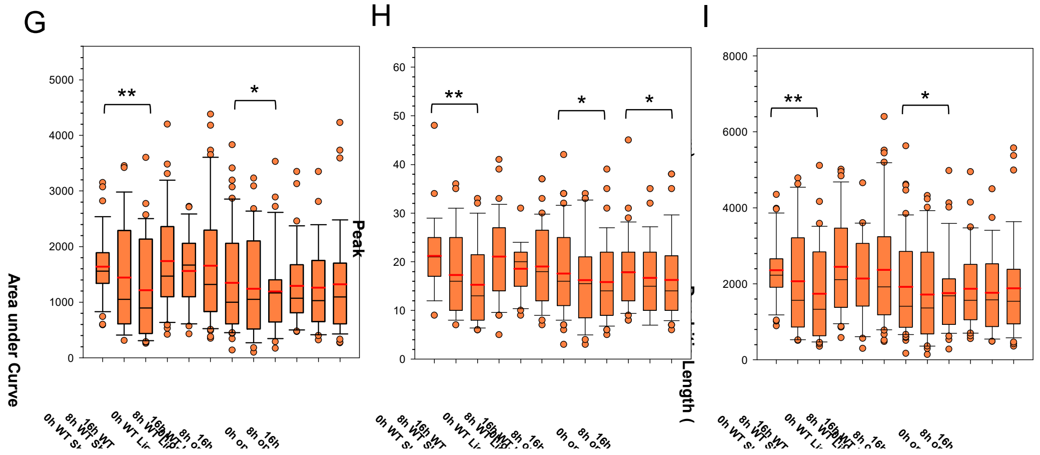

Supplementary Figure S2: Box and whisker plots corresponding to the bar charts shown in Figure 3 G, H and I.

(G) the area under the Sholl curve (AUC); (H) average maximum peak of Sholl curve; and (I) average total dendritic length of the ON-center sub-population of RGCs. All measures revealed significant degeneration over 16 hours in the sham-treated WT and Opa1$^{+/-}$ RGCs. Black line: mean; Red line: median. The ends of boxes correspond to the 25th and 75th percentiles, the whiskers correspond to the 10th and 90th. * *p*<0.05, ** *p*<0.01, ***p<0.001, † *p*<0.05; Mann-Whitney *U* test.
